# Promotion of Tanning Behaviors on Instagram: Content Analysis

**DOI:** 10.2196/110157

**Published:** 2026-10-30

**Authors:** Tailisha Gonzalez, Grace C Hillyer, Bailey Gold, Corey H Basch

**Affiliations:** 1 Department of Health, Administration, Research, and Technology, Lehman College, 250 Bedford Park Blvd W, Bronx, NY, 10468-1588, United States, 1 347-699-6505; 2 Department of Health Studies and Applied Educational Psychology, Teachers College of Columbia University, New York, NY, United States; 3 Department of Public Health, William Paterson University, Wayne, NJ, United States

**Keywords:** Instagram, social media, tanning, skin cancer prevention, content analysis, sun protection, ultraviolet radiation, health communication, adolescent health

## Abstract

**Background:**

Adolescents and young adults may be particularly susceptible to social media content that promotes tanning and appearance-based beauty standards.

**Objective:**

This study examined the characteristics and messaging of pro- and antitanning Instagram posts and the extent to which they promoted health damaging– or health enhancing–behaviors.

**Methods:**

A cross-sectional content analysis of 100 Instagram posts using the hashtag #tanning assessed creator characteristics, tanning practices, commercial promotion, tanning-related messaging, beauty standards, and health risks and benefits. *χ*^2^ and Fisher exact tests were used to determine differences between pro vs anti stances for categorical and dichotomous variables, and the Mann-Whitney *U* test was used for continuous variables.

**Results:**

Most videos were protanning (79/100). Protanning videos were significantly greater than antitanning videos to have been posted in 2026 (77.2% vs 47.6%; *P*=.02), feature teen and young adult creators (87.3% vs 52.4%; *P*<.001), and include shopping tags promoting tanning products (30.4% vs 0%; *P*<.001). Overall, messaging focused on promoting tanning (76%) and advocating for tanned skin as a standard of beauty (63%) and downplayed the use of sun protection (11%) and the risks associated with tanning (20%). Messaging varied significantly by tanning stance, with antitanning videos focused on sun protection and health risk.

**Conclusions:**

Findings indicate an urgent need for clinicians to lead content creation on popular social media platforms to counter the health-damaging effects of unreliable, incomplete, and potentially misdirected information that is posted under popularized hashtags.

## Introduction

Social media increasingly plays a significant role in disseminating content with negative health consequences for adolescents and young adults, especially algorithm-amplified content that promotes Western wealth culture such as tanned skin. Dermatology experts in Australia recently reported a resurgence of dangerous tanning practices driven by influencers who glamorize tan lines and promote SPF-free products [1]. A study of Spanish young adults, all of whom experienced a sunburn in the past year, found that women were more likely to prefer tanned skin after reading tanning-related social media messages despite risk warnings [2]. Public health practitioners have attempted to counter tanning misinformation on social media with health-enhancing information and practices. Despite these efforts, an intervention that educated US young adults on risks associated with indoor tanning failed to detect significant change in salon foot traffic [3]. The impact of social media on health-damaging behaviors and the dissemination of incomplete information or misinformation continues to be documented in real time. The American Academy of Dermatology Association (AADA) conducted a survey of Gen Z adults and found that approximately one-third of respondents used sunscreen only when pressed by others, about 1 in 4 prioritized tanning over skin cancer prevention, slightly more than half were unaware of sunburn risk (eg, skin cancer), and more than 1 in 4 reported skin damage from sun exposure [4,5]. More recently, the AADA warned that social media misinformation has increased skin cancer risk for more than 16 million people in the US, with nearly half of survey respondents reporting that they encountered sunscreen misinformation online; an even greater proportion was reported among Gen Z (64%) [6]. The majority of respondents (83%) believed that tanning contributed to perceptions of good health [6].

Skin cancer is the most prevalent form of cancer, with 70% to 95% of melanomas in fair-skinned people caused by UV radiation [7]. Indoor tanning, which exposes users to UV rays, has been linked to increased incidence of melanoma, basal cell carcinoma, and squamous cell carcinoma, especially among those who started indoor tanning in young adulthood [8,9]. Sunburns have also been linked to increased skin cancer risk [10]. Despite heightened associations with cancer risk, engagement in sun protection behaviors remains low, especially among adults aged 18 to 25 years [11]. Given rising US skin cancer rates, this study examines the characteristics and message content of pro- and antitanning Instagram posts and whether they promote health-damaging or health-enhancing behaviors.

## Methods

Instagram was searched on July 20, 2026, using the hashtag #tanning, which was the most popular tanning-related hashtag on the platform at the time of data collection, sorted by recent posts. The first 100 video posts returned under this hashtag were selected for analysis. Posts were excluded if they were duplicates, non-English, or unrelated to tanning; excluded posts were replaced with the next eligible post in the sequence until 100 videos were retained. A codebook was developed a priori by the study team to capture video characteristics and message content. The video characteristics coded were engagement (views, likes, comments, and shares), year posted, perceived creator age (teen, aged 13‐19 years; young adult, aged 20‐29 years; adult, aged 30+ years; unclear) and gender, setting (tanning bed, outside in full sun, outside in shade, indoors), use of self-tanner or spray tan, before and after images, product promotion (shopping tag, verbal/visual only, none), humor, and peer influence. Message content was coded as promotion of tanning, tanned skin as a beauty standard, maximizing UV index, claims that tanning beds are safe, use of sun protection, health benefits (mental health, skin health, other), shrugging off risks, and dismissing cancer risk. Videos were classified as pro- or antitanning based on their dominant message.

All 100 videos were coded by one trained coder (BG) using this codebook. To assess interrater reliability, a second coder (CHB) independently coded a randomly selected subsample of 10% of the videos. Interrater reliability on the subsample was assessed using the Cohen kappa (κ=0.88), indicating high agreement; discrepancies between coders were resolved through discussion and consensus. The characteristics and message content were examined using frequency distributions, medians, and IQRs. To compare videos with a pro vs anti stance, we conducted univariable analyses with *χ*^2^ or Fisher exact tests for categorical and dichotomous variables and used the Mann-Whitney *U* test for continuous variables. All tests were 2-tailed, with statistical significance set at *P*<.05. All analyses were performed using SPSS statistical software (version 30; IBM Corp).

### Ethical Considerations

This study was reviewed by the William Paterson University Institutional Review Board ([IRB]; protocol number IRB-FY2026-214) and determined to be non–human subjects research. IRB review and approval was not required under 45 CFR 46.102e.

## Results

Results for our analyses of video characteristics are shown in Table 1 and messaging in Table 2. The videos in our sample were viewed collectively over 259 million times. The median number of views per video was 602,000 (IQR 91,250‐2,525,000) and did not differ by stance (Table 1). Overall, videos garnered a median of 79 (IQR 8‐419) comments, with anti videos gathering nearly 8 times as many comments as pro videos (395 [IQR 34‐1164] vs 51 [IQR 5‐269]; *P*=.006). Videos were predominantly shared by female-identified content creators (83/100, 83%) and teens or young adults (80/100, 80%). All protanning videos (79/79) were posted by consumers. Postings of pro videos in 2026 were significantly greater than anti videos (61/79, 77.2% vs 10/21, 47.6%; *P*=0.02). Pro videos were also presented more often by teens and young adults (69/79, 87.3% vs 11/21, 52.4%; *P*<0.001), were more frequently filmed in an indoor setting (29/79, 36.7% vs 1/21, 4.8%; *P*=.002), and more often promoted a tanning product with a shopping tag (24/79, 30.4% vs 0/21, 0%; *P*<.001) compared with anti videos. Pro videos also displayed self-tanner or spray tan more than anti videos (31/79, 39.2% vs 1/21, 4.8%; *P*=.002). Nearly 4 in 10 protanning videos (39.2%) featured self-tanner or spray tan. Of the 21 antitanning videos, 8 (38.1%) were posted by clinicians and 13 (62%) by consumers; none were posted by public health agencies or other health organizations.

**Table 1.** Characteristics of Instagram posts (N=100) related to tanning behaviors by stance.

Video characteristics	Total	Pro (n=79)	Anti (n=21)	*P* value
Views				.08
Median (IQR)	602,000 (91,250-2,525,000)	464,000 (72,600-2,000,000)	1,500,000 (215,000-7,850,000)	
Range	723‐32,600,000	1949‐32,600,000	723‐22,300,000	
Likes				.63
Median (IQR)	8983 (508-56,850)	8371 (459-57,000)	11,600 (2810‐55,450)	
Range	0‐583,000	0‐331,000	5‐583,000	
Comments				.006
Median (IQR)	79 (8-419)	51 (5-269)	395 (34‐1164)	
Range	0‐5641	0‐4178	2‐5641	
Shares				.53
Median (IQR)	997 (61‐9617)	971 (57‐8424)	1022 (142‐16,238)	
Range	0‐879,000	0‐160,000	0‐879,000	
Year posted, n (%)				.02
2020	1 (1)	0 (0)	1 (4.8)	
2024	6 (6)	5 (6.3)	1 (4.8)	
2025	22 (22)	13 (16.5)	9 (42.9)	
2026	71 (71)	61 (77.2)	10 (47.6)	
Perceived creator age, n (%)				<.001
Teen	23 (23)	22 (27.8)	1 (4.8)	
Young adult	57 (57)	47 (59.5)	10 (47.6)	
Adult	17 (17)	7 (8.9)	10 (47.6)	
Unclear	3 (3)	3 (3.8)	0 (0)	
Perceived gender, n (%)				.06
Male	16 (16)	9 (11.4)	7 (33.3)	
Female	83 (83)	69 (87.3)	14 (66.7)	
Unclear	1 (1)	1 (1.3)	0 (0)	
Setting, n (%)				.002
Tanning bed	15 (15)	13 (16.5)	2 (9.5)	
Outside, full sun	55 (55)	37 (46.8)	18 (85.7)	
Outside, shade	0 (0)	0 (0)	0 (0)	
Indoors	30 (30)	29 (36.7)	1 (4.8)	
Self-tanning/spray tan, n (%)				.002
Self-tanner	21 (21)	20 (25.3)	1 (4.8)	
Spray tanner	11 (11)	11 (13.9)	0 (0)	
Neither	68 (68)	48 (60.8)	20 (95.2)	
Before/after images, n (%)				.49
Yes	30 (30)	25 (31.6)	5 (23.8)	
No	70 (70)	54 (68.4)	16 (76.2)	
Product promotion, n (%)				<.001
Shopping tag	24 (24)	24 (30.4)	0 (0)	
Verbal/visual only	15 (15)	13 (16.4)	2 (9.5)	
None	61 (61)	42 (53.2)	19 (90.5)	
Uses humor, n (%)				.30
Yes	28 (28)	24 (30.4)	4 (19)	
No	72 (72)	55 (69.6)	17 (81)	
Peer influence, n (%)				.55
Yes	39 (39)	32 (40.5)	7 (33.3)	
No	61 (61)	47 (59.5)	14 (66.7)	

**Table 2.** Message content of Instagram posts (N=100) related to tanning behaviors by stance.

Messaging	Total	Pro (n=79)	Anti (n=21)	*P* value
Promotes tanning, n (%)				<.001
Yes	76 (76)	75 (94.9)	1 (4.8)	
No	19 (19)	0 (0)	19 (90.5)	
Ambiguous	5 (5)	4 (5.1)	1 (4.8)	
Considers tanned skin a beauty standard, n (%)				<.001
Yes	63 (63)	58 (73.4)	5 (23.8)	
No	37 (37)	21 (26.6)	16 (76.2)	
Maximizing UV index, n (%)				.51
Yes	15 (15)	11 (13.9)	4 (19)	
No	85 (85)	68 (86.1)	17 (81)	
Tanning beds safe, n (%)				.50
Yes	2 (2)	2 (2.5)	0 (0)	
No	10 (10)	7 (8.9)	3 (14.3)	
Unknown	88 (88)	70 (88.6)	18 (85.7)	
Use of sun protection, n (%)				<.001
Yes	11 (11)	3 (3.8)	8 (38.1)	
No	89 (89)	76 (96.2)	13 (61.9)	
Mental health benefit, n (%)				>.99
Yes	6 (6)	5 (6.3)	1 (4.8)	
No	94 (94)	74 (93.7)	20 (95.2)	
Skin health benefit, n (%)				>.99
Yes	2 (2)	2 (2.5)	0 (0)	
No	98 (98)	77 (97.5)	21 (100)	
Other health benefits, n (%)				>.99
Yes	1 (1)	1 (1.3)	0 (0)	
No	99 (99)	78 (98.7)	21 (100)	
Shrugging off risks, n (%)				.006
Yes	20 (20)	20 (25.3)	0 (0)	
No	80 (80)	59 (74.7)	21 (100)	
Dismisses cancer risk, n (%)				.34
Yes	6 (6)	6 (7.6)	0 (0)	
No	94 (94)	73 (92.4)	21 (100)	

Of the 100 posts analyzed, the messaging focused on promoting tanning (76, 76%) and advocating for tanned skin as a standard of beauty (63, 63%) and downplayed the use of sun protection (11, 11%) and the risks associated with tanning (20, 20%) (Table 2). While protanning videos heavily promoted tanning (75/79, 94.9% vs 1/21, 4.8%; *P*<.001) and considered tanned skin a standard of beauty (58/79, 73.4% vs 5/21, 23.8%; *P*<.001), anti videos more often emphasized the use of sun protection (8/21, 38.1% vs 3/79, 3.8%; *P*<.001) and did not shrug off overall risks associated with tanning and UV exposure (21/21, 100% vs 59/79, 74.7%; *P*=.006). Messages promoting mental health, skin health, or other health benefits of tanning did not differ significantly by stance.

## Discussion

Within this sample, Instagram content under the #tanning hashtag disproportionately promoted tanning behaviors without including reliable information about health risks (eg, skin cancer, long-term UV radiation exposure). Sunless tanning is considered a safer alternative to UV exposure, but these videos still promoted tanned skin as a beauty standard and rarely mentioned sun protection. Sunless tans do not protect against UV damage, and spray tans carry their own risks, such as inhaling the product [12]. Research by Seidenberg and colleagues [13] highlights additional risks with sunless tanning. Nearly 1 in 4 women aged 18 to 49 years used both sunless tanning products and indoor tanning, potentially compounding their skin cancer risk [13]. Additional research is needed on co-occurring tanning practices within this population.

A hashtag that garners over 250 million views is considered a high-performing topic; thus, there were multiple lost opportunities to steer users interested in tanning toward health-promoting behaviors. A 2021 review of articles on social media and skin cancer awareness highlighted the positive role dermatologists play in disseminating reliable health information, indicating increased participation by these health care professionals across social media platforms [14]. Unfortunately, the current study found clinician content creators largely absent. Clinicians created 8/21 (38.1%) of the antitanning videos, while all other videos were created by consumers. Clinician videos reached a sizable audience (median 1.25 million views), but the messages with protanning content they were competing with outnumbered them. Most videos promoted tanning as a beauty standard and were dominated by teens and young adults, the majority of whom did not promote any type of sun protection and sometimes actively shrugged off health risks. This disconnect highlights the need for health care professionals to routinely engage in skin cancer prevention, given the understanding that social media runs on platforms that reach new users continuously. Previous research has demonstrated a disconnect between video popularity and the educational value of short-video platform content hosted on sites such as Instagram. The content produced by clinicians may be of greater quality and indicate credibility, but low interaction metrics with audiences indicate a “quality popularity paradox” [15].

Experts previously outlined the ethical implications of social media algorithms that promote high-risk tanning practices and normalize preventable harm [16]. Adolescents and teens are especially vulnerable to appearance-based messaging, risk-taking, and immediate cosmetic reward [16]. More research is required to explore health information posts that emulate successful content creators’ familiarity with social media algorithms [17]. There are many facets that continue to surface, such as greater availability of information in video titles vs descriptions, emotional intensity of videos, and “drama,” or the ability to stimulate conflict among viewers [17,18]. Maximizing these platforms for health communication requires employing hashtags that demonstrate relevance to the audience’s interest instead of the clinician’s (eg, #tanning vs #skincancer) [19].

Experts previously outlined the ethical implications of social media algorithms that promote high-risk tanning practices and normalize preventable harm [16]. Adolescents and teens are especially vulnerable to appearance-based messaging, risk-taking, and immediate cosmetic reward [16]. More research is required to explore health information posts that emulate successful content creators’ familiarity with social media algorithms [17]. There are many facets that continue to surface, such as greater availability of information in video titles vs descriptions, emotional intensity of videos, and “drama,” or the ability to stimulate conflict among viewers [17,18]. Maximizing these platforms for health communication requires employing hashtags that demonstrate relevance to the audience’s interest instead of the clinician’s (eg, #tanning vs #skincancer) [19].

There is an urgent need for clinicians and organizations promoting sun protective behaviors and products to lead content creation on popular social media platforms. Clinicians are positioned to counter the health-damaging effects of unreliable, incomplete, and potentially misdirected information that is posted under popularized hashtags. Commercial enterprises can compound the effects of this messaging by promoting sun-protective clothing, shade devices, hats, sunscreen, UV detectors, and sunglasses. This is a unique and timely opportunity for collaboration across these sectors to counter the damaging impacts of protanning messaging. The need for public-private partnerships may address the null effect some public health practitioners have experienced in implementing tanning interventions via social media platforms [3]. Our study findings support the need for more sustained skin cancer prevention campaigns targeting tanning behaviors [20].
